# Opioid drug seeking after early-life adversity: a role for delta opioid receptors

**DOI:** 10.1016/j.addicn.2024.100175

**Published:** 2024-09-19

**Authors:** Sophia C. Levis, Matthew T. Birnie, Yiyan Xie, Noriko Kamei, Puja V. Kulkarni, Johanna S. Montesinos, Christina R. Perrone, Catherine M. Cahill, Tallie Z. Baram, Stephen V. Mahler

**Affiliations:** a Department of Anatomy & Neurobiology, University of California Irvine, Irvine, CA, USA; b Department of Neurobiology & Behavior, University of California Irvine, Irvine, CA, USA; c Department of Pediatrics, University of California Irvine, Irvine, CA, USA; d Department of Psychiatry and Human Behavior, University of California Irvine School of Medicine, Orange, CA, USA; e Department of Psychiatry and Biobehavioral Sciences, Shirley and Stefan Hatos Center for Neuropharmacology, Semel Institute for Neuroscience and Human Behavior, David Geffen School of Medicine, University of California Los Angeles, Los Angeles, CA, USA.

**Keywords:** Early life adversity, Opioid addiction, Delta opioid receptor, Demand elasticity, Nucleus accumbens

## Abstract

Opioid use disorder (OUD) is associated with a history of early-life adversity (ELA), an association that is particularly strong in women. In a rodent model, we previously found that ELA enhances risk for opioid addiction selectively in females, but the mechanisms for this effect are unclear. Here, we show that ELA robustly alters cFos responses to opioid drugs in females’ nucleus accumbens (NAc) and basolateral amygdala (BLA), but not else-where. We further identify delta opioid receptors (DOR), which mature in the first week of life and thus later than kappa or mu opioid receptors, as a potential mediator of ELA’s impacts on reward circuit functions. Accordingly, DOR mRNA in NAc was persistently reduced in adult females with ELA history. Moreover, pharmacological stimulation of NAc DORs increased opioid demand in control females (recapitulating the ELA phenotype), while blocking DORs in ELA females conversely reduced high-effort drug consumption, simulating the control rearing phenotype. These findings support a role for NAc DORs in mediating ELA-induced opioid vulnerability. In contrast, BLA neurons expressing DOR protein do not overlap heroin- responsive cells in ELA rats, arguing against a direct relationship of BLA DORs to heroin’s addiction-relevant actions in the brain. Together, these results suggest a novel and selective role for NAc DORs in contributing to enduring, ELA-provoked vulnerability to OUD.

## 1 Introduction

Early life adversity (ELA) is a common risk factor for the development of substance use disorders, including opioid use disorder (OUD). Because addictive behaviors are mediated in part by brain reward circuits, these observations suggest that ELA may disrupt reward circuit function [1–21]. Intriguingly, this association appears to be particularly strong in women [6,9,22–27]. In rats and mice, we and others have accordingly found that a limited bedding and nesting model of ELA causes long term changes in reward behaviors and reward circuit function in both sexes [13,14,16,18,19,28–32], and may impose risk for opioid addiction uniquely in female rodents [14,18–20,28]. However, the specific molecular and circuit-level changes, especially within the endogenous opioid system, that underlie ELA-induced behavioral phenotypes remain unclear.

The endogenous opioid system plays an essential role in goal-directed behaviors including the rewarding effects of addictive drugs [33]. Although there has been a significant emphasis on mu opioid receptors, which within mesolimbic circuits are necessary and sufficient for opioid drug reward [34–36], kappa and delta opioid receptors (DORs) also importantly regulate reward seeking behavior [37–46]. Since changes in endogenous opioid receptor systems likely contribute to drug-seeking behavior, and since ELA is a well-established risk factor for addiction (perhaps especially in women), we asked whether ELA imposes vulnerability to opioid addiction in female rodents via modifications of endogenous opioid signaling that alter brain responses to acute or chronic exposure to opioid drugs.

First, we determined whether ELA alters brain responses to heroin, either as a first exposure to the drug, or following chronic opioid self-administration. We quantified cFos expression after heroin in reward-and stress-related brain regions that have previously been implicated in mediating effects of ELA [20,47–52], namely nucleus accumbens (NAc), amygdala, prefrontal cortex, and paraventricular thalamus, as well as in neurons projecting to NAc. We identified that ELA alters brain responses to heroin, especially in NAc. Pursuing the brain mechanisms responsible for altered neuronal activity in ELA rodents, we next investigated how ELA affects opioid receptor expression. We examined the developmental trajectory of opioid receptor mRNA expression in NAc and amygdala, where we identified that delta opioid receptors mature later than mu or kappa receptors and thus might be especially vulnerability to disruption by ELA. Consistent with this possibility, NAc delta opioid receptor mRNA levels remained reduced into adulthood by early life adversity.

Finally, to determine to what extent ELA may alter DOR signaling, we pharmacologically manipulated DORs in NAc in ELA- and control-reared females, and examined the effects on drug seeking using an intravenous self-administration behavioral economic demand elasticity task. We found that DOR manipulations altered motivation for opioid drugs distinctly in ELA and CTL rats, indicating that aberrations in NAc DOR signaling may be a risk factor in the development of OUD following ELA in females. Taken together, our findings support a role for DORs in limbic modulation of opioid reward, and in the susceptibility to opioid addiction induced by ELA.

## 2 Materials and methods

### 2.1. Animals

Food and water were available *ad libitum* throughout all experiments. All procedures were approved by the University of California Irvine Institutional Animal Care and Use Committee and conducted in accordance with the National Institutes of Health guide for the care and use of laboratory animals.

Rats: Primiparous, timed-pregnant Sprague-Dawley rats were obtained from Envigo (Livermore, CA) on gestation day 15, and maintained in an uncrowded, quiet animal facility room on a reverse 12-hr light/dark cycle. Parturition was checked daily, and the day of birth was considered postnatal day (PD) 0. On PD2, litters were mixed, and pups and dams were assigned to ELA and control (CTL) groups and housed under these conditions through PD9, as described below. Males (n = 6 CTL / 11 ELA) and females (CTL n=48, ELA n=50) were used for these experiments. Rats were weaned at PD21, and thereafter housed by sex in groups of 2-3 males and 2-4 females, under a 12-hr reverse light cycle. Rats remained undisturbed until behavioral testing began at approximately PD60.

Mice: To measure opioid receptor mRNA expression across the life-span, control c57bl/6 female mice bred in-house, group housed under standard conditions on a standard 12-hr light cycle, were sacrificed on either near the end of the ELA period tested here (PD10; n = 4) or at adulthood (PD60; n = 10), for region-specific PCR mRNA quantification.

### 2.2. Drugs

Heroin (diacetylmorphine) HCl was provided by the National Institute on Drug Abuse (NIDA) Drug Supply Program (Research Triangle Park, NC, USA) or Cayman Chemical Company (Ann Arbor, MI, USA), and remifentanil HCl was provided by the NIDA Drug Supply Program. The DOR antagonist naltrindole hydrochloride (NTI), and the DOR agonist [D-Pen [2,5]]-Enkephalin hydrate (DPDPE) were obtained from Sigma-Aldrich (St Louis, MO, USA). Heroin and remifentanil were dissolved in sterile 0.9% saline, DPDPE was dissolved in sterile artificial cerebrospinal fluid (ACSF), and NTI was dissolved in sterile water for intracranial microinjection.

### 2.3. Limited Bedding and Nesting (LBN) early-life adversity protocol

On PD2, rat pups from at least two litters at a time were gathered and assigned at random to each dam, with equal numbers of male and female pups, to prevent confounding effects of genetic variables, sex ratios, or litter size (n=10 pups per dam, with no pups rejected by dams). Dams were then assigned in roughly equal number to LBN or CTL rearing cages, as described previously [18,19,53,54]. Cages remained undisturbed during PD2-9. Throughout this epoch, maternal behaviors, which may constitute a source of stress in infant rats, were video monitored and quantified, as previously described [53,55,56]. On PD10, both ELA and CTL animals were transferred to standard (CTL) cages, which results in rapid normalization of maternal behaviors, and dissolution of stress in pups [55].

### 2.4. Assessing reward circuit activation by heroin

#### 2.4.1. NAc projection tracing

Opioid-experienced female rats that had undergone previously published opioid self-administration training [28] (n = 9 CTL / 8 ELA) and handled, age-matched, opioid-naïve females (n= 7 CTL / 7 ELA) received stereotaxic unilateral NAc pressure injections of ∼40 nL of cholera toxin beta subunit (CTb, 0.5%) aimed at the border of NAc core and shell (mm from bregma: AP + 1.35; ML + or −1.55; DV −7.6), as previously described [30]. Rats were anesthetized with ketamine/xylazine (66/8mg/kg, i.p.). Meloxicam (1mg/kg, i.p.) for postsurgical analgesia, and prophylactic antibiotic cefazolin (0.2ml, i.m.; 10mg/0.1ml) were administered intraoperatively. They were allowed to recover for a minimum of 5-7 days prior to an acute heroin challenge and the resulting cFos expression experiments (described below), a duration that produces adequate CTb expression does not appear to affect cFos detection [57–59].

#### 2.4.2. Heroin-associated neuronal activation

A minimum of 5 days following CTb surgeries, rats were injected with heroin (0.25mg/kg, s.c.) and immediately placed in a novel environment (43 × 43 × 30.5cm chamber with transparent walls and without bedding or food/water) for one hour, then returned to home cages. This non-sedating dose and route of administration was chosen due to its reinforcing and reinstating properties [28,30,60]. Animals were sacrificed via transcardial perfusion 120 minutes following the heroin injection for cFos protein analysis. Following sacrifice, tissue was postfixed, and frozen 40-µm coronal sections were collected for cFos and CTb quantification. CTb injection sites were confirmed using fluorescent immunolabeling, as previously published [30]. Most injections spread to both shell and core of NAc. Brains with misplaced CTb injections or leakage beyond NAc borders were not used for CTb quantification and only used for quantifying cFos expression. Sixteen opioid-experienced (8 ELA / 8 CTL) and 11 opioid-naïve (6 ELA / 5 CTL) had adequate CTb placements and were included in tracer experiments. The remaining rats were excluded from tracer analyses due to misplaced CTb. PVT sections of one opioid-experienced ELA rat and PFC sections of one opioid-naïve CTL rat were excluded analyses due to tissue damage.

An avidin-biotin complex (ABC)-amplified, diaminobenzidine (DAB) reaction was conducted to visualize heroin-induced cFos and CTb expression. Tissue collection, preparation, and cFos and CTb immuno-histochemistry were performed according to previously published methods [30]. Initial cFos staining procedures were conducted using Millipore polyclonal rabbit anti-cFos (#ABE457; 1:5,000), however due to stock shortages, we later employed Abcam’s polyclonal rabbit anti-cFos antibody (#GR3293718-1, 1:10,000). The two antibodies were previously compared head-to-head, and did not differ significantly [30], so data from both antibodies were combined.

#### 2.4.3. Imaging and cFos analysis

Images of structures quantified for cFos/CTb were taken at 10X magnification on a Leica DM4000B microscope with stage automation and stitched using Stereo Investigator (SI) software (MicroBrightfield). Three to four coronal sections per structure from comparable regions in each animal were quantified bilaterally by a trained, blinded observer. Counts from these sections were averaged for each animal, and this average was used for statistical analysis. Brain region borders were delineated based on a brain atlas (Paxinos and Watson, 2007). The coordinate range sampled from each structure is as follows (mm relative to bregma): PFC: +3.24 - +3.00; NAc: +2.28 - +1.44; BLA: −2.28 - −2.92; aPVT: −1.32 - −2.28; pPVT: −3.14 - −4.16. NAc and PFC were further delineated into sub-structures (NAc core and shell and infralimbic and prelimbic PFC). cFos+ neurons were identified using the SI particle counter tool [30], and cFos density (Fos/mm^2^) was computed for each sample as in our prior reports [18]. On sections also stained for CTb, the SI particle counter tool was used only on the hemisphere contralateral to the injection site and manually checked by a trained observer to avoid misidentification of cells. CTb+ only, cFos+ only, and dual-labelled (CTb+ and cFos+) neurons were quantified manually in ImageJ from the hemisphere ipsilateral to the CTb injection. To normalize variability in precise CTb injection volume and localization across animals, the percentage of NAc-projecting (CTb+) cells that were also cFos+ was used for primary analyses [61,62].

### 2.5. Assessing effects of ELA on opioid receptor expression

#### 2.5.1. Tissue micro-punching

Frozen whole brains from 34 female (opioid-naïve: 11 CTL / 9 ELA; opioid-experienced: 8 CTL / 6 ELA), and 17 opioid-naïve male rats (6 CTL / 11 ELA) were brought to −10°C in a cryostat (CM3050S, Leica Biosystems, Germany). Structure locations were identified from an atlas (Paxinos & Watson, 2006), and brains were sectioned coronally into 200µm serial sections using the following coordinate ranges (mm from bregma): mPFC 3.7 – 2.2, NAc 1.7 – 0.7, BLA −1.8 – −3.3, aPVT −1.3 – −2.3, pPVT −3.1 – −4.2. A single punch (Uni-Core, Whatman Harris, US) was taken from each section, targeted at the center of midline mPFC (1 × 2mm), aPVT (1 × 1mm) and pPVT (1 × 1mm) regions, and bilaterally for BLA (2 × 1mm) and NAc (2 × 1mm).

To assess effects of ELA on opioid receptor expression through development, infant (P10) and adult (P60) mice were used in lieu of rats due to technical limitations. In adult experimentally-naïve (CTL) female mice, BLA and NAc were extracted using the Palkovits Punch technique [63]. Briefly, BLA coordinates were identified from an atlas (Paxinos & Watson, 2006), and were sectioned coronally into 200µm serial sections using the following coordinate ranges (mm from bregma): BLA −0.7 – −2.54, NAc 1.94 – 0.74. Bilateral 1mm punches (Uni-Core, Whatman Harris, US) were taken from each of eight BLA sections and five NAc sections, stored in a 1.5mL tube (Eppendorf, Germany). In PD10 mice, brains were sectioned into 200 µm sections, and serial sections (3 × 200µm for NAc; 4 × 200µm for BLA) and bilateral 1mm punches were taken. Structures were identified using anatomic landmarks (based on Paxinos & Watson, 2006). Punches were stored on dry ice until RNA extraction.

#### 2.5.2. Reverse-transcription quantitative PCR

Total RNA was extracted from each tissue punch using the Direct-zol RNA preparation kit (Zymo Research). Complementary DNA was prepared from 100 ng of DNAse-I treated RNA using the Transcriptor firststrand cDNA synthesis kit (Roche) with oligo d(T) and random hexamer primers.

Primer set sequences for μ, κ, and δ opioid receptor transcripts (*oprm1, oprk1, and oprd1,* respectively) are listed in Supplementary Table 1. Reverse transcription quantitative PCR was performed with SYBR green (FastStart Essential DNA Green Master; Roche) and run in triplicate on a Roche Lightcycler 96 system. Data were quantified using the 2^-ΔΔ^*C*_T_ method, using 18S rRNA as internal control. Samples with values meeting outlier criteria (Grubbs’ alpha < 0.05) were excluded.

#### 2.5.3. Delta opioid receptor fluorescent immunolabeling and quantification

Neighboring NAc and BLA sections from rat brains used for tracer studies above were fluorescently labeled for cFos and delta opioid receptors. Sections were incubated in rabbit anti-DOR (1:20k; #RA19072, Neuromics, Edina, MN; validated in dorsal root ganglia neurons [64]) for 10 days at 4°C, washed, then incubated in guinea pig anti-cFos [65, 66] (1:5k; #226 005, Synaptic Systems; Goettingen, Germany) overnight at room temperature. After washing, sections were incubated in donkey anti-guinea pig Alexa Fluor 594 (1:500; Jackson ImmunoResearch, West Grove, PA) and donkey anti-rabbit Alexa Fluor 488 (1:500; Fisher/Invitrogen, Waltham, MA) at room temperature for 4 hours and washed. Finally, sections were washed with DAPI nuclear stain, mounted, and coverslipped with Fluoromount mounting medium and stored at 4°C until photographed. Images of BLA from comparable regions in each animal (coordinate range, mm from bregma: −2.52 – −3.12, Paxinos and Watson, 2007) were taken at 40X magnification on a Leica SP8 confocal microscope. For each section, the entire BLA was delineated at 10x magnification based on anatomic and morphologic landmarks (ie. external capsule laterally, distribution of DAPI+ cells medially and inferiorly) then imaged at 40X magnification with automated stitching. Z-stacks of 8 slices each were acquired for each 40µm thick section, and were transformed into a maximum intensity projection 2D image using ImageJ software prior to quantification. Quantification was performed by a trained, blinded observer. DOR+, cFos+, and double-labeled neurons were quantified manually using ImageJ, and DAPI+ cells were quantified using the particle analyzing tool in ImageJ. To account for variability in area of the region quantified, overall cell density, and tissue quality, the number of DOR+ cells were quantified both as a proportion of the total number of DAPI+ cells in the region and by the density of cells per square millimeter. Somatic DOR labeling was not observed in immunolabeled NAc sections at 10x or 40x magnifications, therefore DOR and cFos were not quantified in NAc.

### 2.6. Assessing the effects of DOR activation and blockade on opioid reward

#### 2.6.1. Intravenous catheter surgery

At approximately PD60, ELA and CTL rats were deeply anesthetized with isoflurane (2-2.5%) and sterilized chronic indwelling catheters made in-house were inserted into the right jugular vein. Meloxicam and cefazolin were administered intraoperatively, as above. After 5 days of recovery, rats began self-administration training, and catheters were flushed daily following each opioid self-administration session with cefazolin (10mg/0.1ml) and heparin solution (10 U/0.1ml) to maintain catheter patency.

#### 2.6.2. Intracranial surgery

Following drug self-administration training (described below) and prior to pharmacologic testing on the behavioral economic task, rats were implanted with bilateral guide cannulae directed at the medial shell of the nucleus accumbens (n = 22 CTL / 23 ELA; 26GA, 2mm center-to-center, Plastics One; coordinates, mm from bregma, after incisor bar is raised +5mm from Z=0: AP +3.0, ML ± 1.0, DV −5.5). Cannulae were attached to the skull using dental cement and four jeweler screws. Rats were anesthetized with ketamine/xylazine and perioperative medications administered as above, and were allowed to recover for 5 days before restarting behavioral testing.

#### 2.6.3. Behavioral economic thresholding procedure

Drug self-administration training and testing took place in Med Associates operant chambers in sound-attenuating boxes, as described previously [28,67–70]. To facilitate learning on the thresholding task, female rats (n = 20 CTL / 23 ELA) were first trained to self-administer remifentanil (3.2ug/kg/infusion) on a fixed ratio 1 (FR1) schedule for a minimum of three days, or until acquisition criteria (>20 infusions in a 2hr session for at least two consecutive days) were reached. They received daily 2-hr self-administration sessions, when pressing on the “active” lever (AL) yielded a remifentanil infusion accompanied by concurrent 2.9-kHz tone and lever light illumination for 3.6s. A 20s timeout period (signaled by turning off the house light) followed each infusion/cue presentation, during which additional lever presses were recorded but had no consequence. Pressing on the second “inactive” lever (IL) was recorded but had no consequence with no cue light or tone. ELA and CTL animals did not differ in the number of training days (mean CTL = 2.7, ELA = 2.6; t_(43)_=0.45, P = 0.65) or the total number of infusions during FR1 training (mean CTL = 143.9, ELA = 171.2, t_(43)_=1.24, P = 0.21).

Once acquisition criteria were reached, rats begun training on a previously described within-session economic thresholding procedure [18,28,30,67,69–72]. As in the FR1 procedure, each AL press delivered remifentanil and a concurrent light and tone cue presentation. The duration of each cue/infusion (and hence the amount of drug per infusion) was decreased in successive 10-min bins across the 110-min session, meaning that rats were required to exert increasing effort (ie. pay a higher price) to obtain their desired blood levels of drug. The resulting doses during each bin are as follows: 2, 1, 0.6, 0.3, 0.2, 0.1, 0.06, 0.03, 0.02, 0.01, 0.006 µg per lever press/infusion [28,67]. Drug intake was determined at each response requirement and consumption data was modeled with an exponential demand equation: lnQ = lnQ_0_ + *k*^(*e*-∝*Q*_0_*C*^ −1) [19,28,67,70,72] where Q=consumption, C=unit cost, k is a scalar constant for consumption range, α = demand elasticity, and Q_0_ = extrapolated intake at zero effort. This process yields a demand curve fitted to consumption at each bin, from which variables corresponding to hedonic set point (Q_0_, reflecting hedonic value of remifentanil extrapolated to price 0) and motivation (α, reflecting demand elasticity or sensitivity to increasing price) are derived. As in prior reports using this approach [18,30,67–70], the highest-effort bins, in which behavior was sporadic or absent, were removed to improve demand curve fitting.

#### 2.6.4. Pharmacologic manipulation of reward motivation

Female rats were trained on the thresholding behavioral economic task for five days prior to undergoing intra-cranial cannulation surgery (described above). After post-operative recovery for 5 days, rats were retrained on the threshold procedure for a minimum of five days and until responding was stable (α and *Q*_*0*_ values within <25% of each other for three consecutive days), and pharmacological testing began once stability criteria were reached. Prior to the first test, rats were acclimated to the microinjection procedure: they were gently restrained, and injectors were lowered into NAcS bilaterally (33GA, Plastics One, protruding 2 mm below cannulae tip) and then removed; no injection was made. The following day, rats underwent the first in a series of tests following pretreatment with counterbalanced intra-NAc microinjections. Five minutes before each such test session, rats received a bilateral infusion of the delta opioid receptor agonist, DPDPE [0.31, or 3.1 µg/0.5µL/side [42,73]], the delta opioid receptor antagonist NTI [1.0 µg/0.5µL/side [45,74,75]], or vehicle [0.5µL/side aCSF for comparison with DPDPE, sterile water for comparison to NTI; all microinjections infused over 75s]. Rats were re-stabilized on the thresholding procedure for at least two days between tests. Two CTL rats did not complete behavioral testing due to intracranial cannula failure during training. Rats that failed to reach stability criteria following cannula surgery (n= 4 CTL / 1 ELA) were excluded from testing and analyses. Four ELA rats were excluded post-hoc due to misplaced cannula outside NAc.

### 2.7. Analytic Approaches

To determine the effects of ELA on heroin-induced cFos expression, opioid receptor mRNA and protein expression, and economic demand characteristics, we used independent samples t-tests. To determine the interactions of ELA and opioid experience on these measures, we used between-subjects two-way ANOVAs. A 3-way ANOVA was used to test interactions of ELA, opioid experience, and NAc shell subregion on cFos expression. Sidak post-hoc tests were used to characterize the nature of significant ANOVA interactions. In ontogeny experiments, average mRNA expression at PD10 was normalized to 1 for each target, and PD60 expression transformed to represent fold-change from PD10. Unpaired t-tests were used to test differences between PD10 and PD60 expression for each mRNA target. One-way ANOVA was used to test whether the change in expression by PD60 differed between mRNA targets. Significant ANOVAs were followed up with Sidak post-hoc tests. In pharmacologic manipulation experiments, to determine the effect of delta opioid receptor agonist or antagonist on drug consumption at different levels of effort (“price”) in ELA and CTL rats, we used 2-way repeated measures ANOVAs with price and drug dose as independent variables. To test effects of DOR agonist (DPDPE) in ELA and CTL rats on demand characteristics at 3 doses (VEH, low dose, high dose), we used a repeated measures one-way ANOVA and followed up significant effects with Dunnett’s post-hoc tests. Because we only used a single dose of NTI and vehicle, we used repeated measures t-tests to determine effects of antagonist on demand characteristics (α and *Q*_*0*_) in ELA and CTL animals. To identify interactions between rearing conditions and DOR manipulation on demand characteristics, we used two-way ANOVAs with drug dose and rearing condition as independent variables. Significant interactions were followed up with Sidak post-hoc tests. Groups did not statistically differ from one another in variance, accommodating assumptions of the parametric tests employed. Statistical analyses were performed using GraphPad Prism software.

## 3. Results

### 3.1. Effects of ELA on reward circuit activation in response to heroin

We previously discovered that ELA drastically modified reward behaviors in response to opioids, leading to an opioid addiction-like behaviors in female rodents [28]. Therefore, here we sought to identify whether ELA altered heroin-induced neuronal activation within reward circuit nodes of animals with or without prior opioid self-administration experience (referred to here as opioid-experienced and opioid-naïve, respectively).

**Fig. 1.**
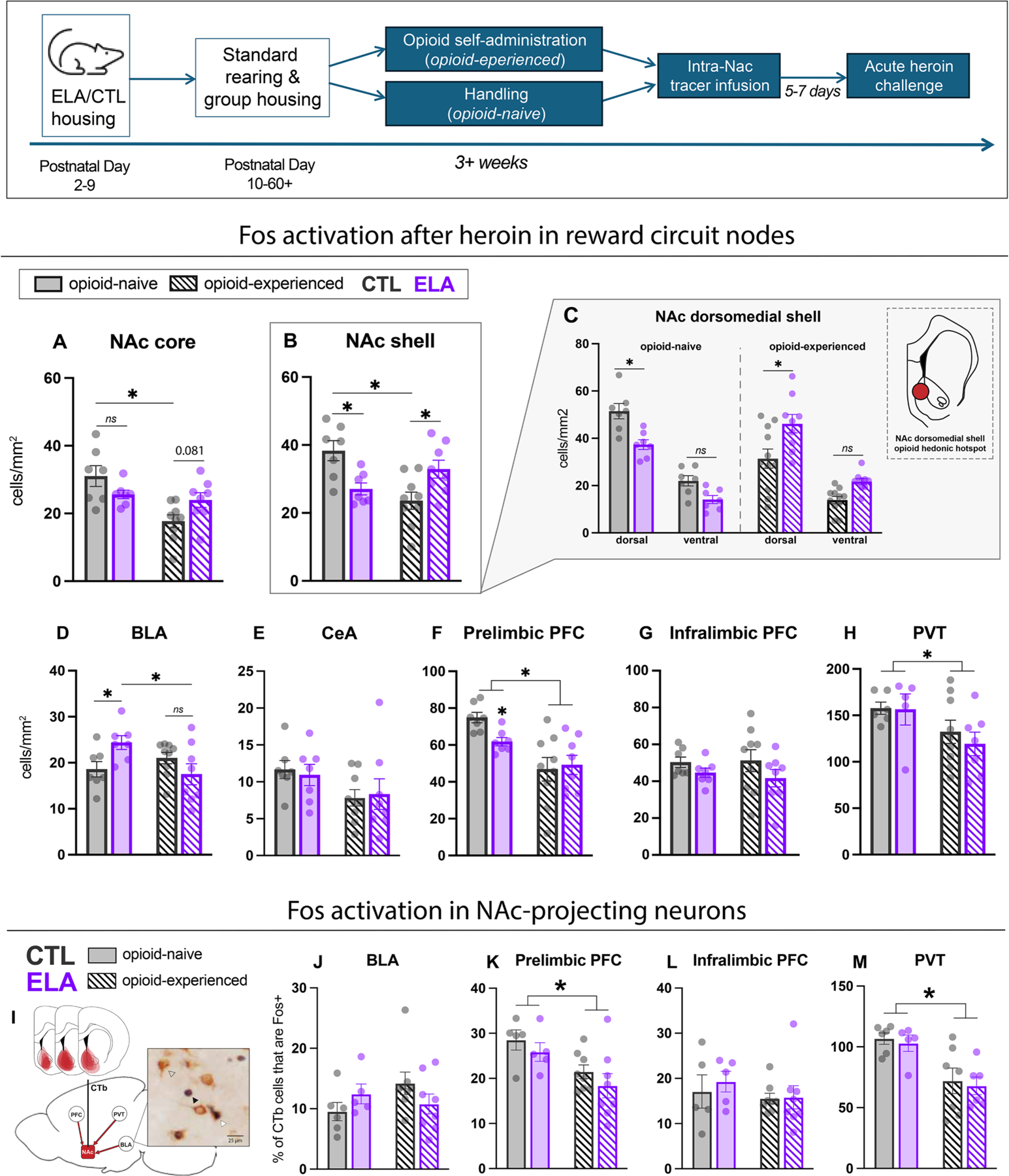
Heroin-induced neuronal activation is influenced by ELA in an opioid experience dependent manner. Average density of cFos+ cells in nucleus accumbens, amygdala, prefrontal cortex, and paraventricular thalamus, and proportion of NAc-projecting neurons with cFos expression in BLA, PFC, and PVT. (A, B) chronic opioid experience reverses ELA blunting of NAc core and shell response to heroin. (C) These effects are most strongly pronounced in the NAc dorsomedial shell, an area containing a previously-defined opioid “hedonic hotspot”. (D-E) ELA increases BLA activation by heroin, an effect that is ameliorated by chronic opioid experience. This affect is unique to the basolateral nucleus of the amygdala: ELA did not alter cFos expression in the central nucleus of the amygdala (CeA). (F) ELA reduced activation by heroin in prelimbic cortex of opioid naïve animals, and chronic opioid experience reduced activation by heroin only in CTL rats. (G) Infralimbic PFC response to heroin was not affected by ELA or opioid experience. (H) Chronic opioid experience reduced activation of PVT but there were no effects of ELA in this region. (I) Diagram of NAc CTb injection placements and example of cell appearance. Black arrow = Fos+, clear arrow = CTb+; white arrow = Fos+CTb+ cell. (J) ELA tended to bidirectionally affect NAc-projecting BLA cells (interaction∼0.08). (K,L) Opioid experience reduced activation of NAc-projecting prelimbic but not infralimbic PFC cells. (M) Opioid experience reduced activation of NAc-projecting cells in PVT and there was no effect of ELA on cFos expression. * P<0.05

To determine effects within the NAc, we delineated its functionally distinct NAc shell and core subregions [42,76–79]. In both NAc subregions, the effect of ELA on heroin-induced neuronal activation varied depending on prior opioid history (Fig. 1A-B). In the NAc shell, opioid-naïve ELA rats had lower cFos expression than controls, whereas opioid-experienced ELA rats had higher cFos expression than their control counterparts following acute administration of heroin (opioid experience x rearing condition interaction F_(1,27)_ =16.33, P<0.001; main effect of opioid experience F_(1,27)_=3.064, P=0.091; rearing condition F_(1,27)_ = 0.1424, P=0.71. Sidak post-hoc: naïve ELA vs CTL t_(27)_=2.985, P=0.012, experienced ELA vs CTL t_(27)_=2.723, P=0.022). Furthermore, opioid experience decreased heroin-associated activation only in CTL animals (Sidak post-hoc t_(27)_=4.151, P=0.0006), whereas chronic opioid experience did not significantly affect NAc activation in ELA rats (Sidak post-hoc t_(27)_=1.598, P=0.23). A similar interaction between ELA and opioid experience was observed in the NAc core (opioid experience x rearing condition F_(1,27)_=7.252, P=0.012; opioid experience F_(1,27)_=11.90, P=0.0019; rearing condition F_(1,27)_=0.0371, P=0.85). While opioid-naïve ELA rats did not differ significantly from controls in NAc core responses to heroin (Sidak post-hoc t_(27)_=1.689, P=0.19), opioid-experienced ELA females had a trend towards higher cFos expression compared to controls (Sidak post-hoc t_(27)_=2.145, P=0.081), with opioid experience significantly reducing cFos expression only in CTL rats

To dive deeper into subdivisions of the shell based on sub-region differences in reward responses [42], we examined the NAc medial shell dorsal and ventral subregions to determine whether changes in neuronal activation were localized specifically to the dorsal region of the medial NAc shell, an area previously found to contain an opioid “hedonic hotspot” (Fig. 1C) [42]. The effects of ELA and opioid exposure were most prominent in the dorsomedial NAc shell rather than the ventromedial region. There was a significant 3-way interaction between rearing condition, opioid experience, and medial shell subregion (F_(1, 30)_=6.598, P=0.015), significant 2-way interactions between opioid experience and subregion (F_(1,30)_=4.295, P=0.047) and opioid experience and rearing condition (F_(1,30)_=18.13, P=0.0002), and a significant main effect of shell subregion (F_(1,30)_=339.2, P<0.0001). In post-hoc analysis, the effects of ELA were significant only in dorsomedial shell, and the comparisons within ventromedial shell were not significant (Sidak post-hoc, *dorsal*: opioid-naïve ELA vs CTL t_(60)_=3.167, P=0.029; opioid-experienced ELA vs CTL t_(60)_=3.931, P=0.0027; *ventral*: opioid-naïve ELA vs CTL t_(60)_=1.766, P=0.64; opioid-experienced ELA vs CTL t_(60)_=2.080, P=0.40).

In addition to assessing changes in neuronal activation in the NAc, we also asked if similar effects might be observed in reward-related regions that project to NAc, including amygdala, prelimbic and infralimbic prefrontal cortex (PFC), and the paraventricular thalamus (PVT). In contrast to NAc, ELA rats without prior opioid experience had greater cFos expression in the basolateral nucleus of the amygdala (BLA) after acute heroin compared to controls (Fig. 1D; t_(12)_=2.593; P=0.024), an effect that was not present in the chronic opioid exposure group (t_(15)_=1.403; P=0.18). To determine potential interactions between ELA and opioid history on BLA responses to acute heroin, we also analyzed these data with a two-way ANOVA (interaction: F_(1,27)_=7.363, P=0.012; main effect rearing condition: F_(1,27)_=0.4386, P=0.51; main effect opioid experience: F_(1,27)_=1.664, P=0.21). Indeed, chronic opioid experience led to reduced cFos expression only in ELA rats (Sidak’s post-hoc t_(27)_=2.794, P=0.019), and did not appear to alter BLA responses to heroin in CTL rats (post-hoc t_(27)_=1.020, P=0.53). The effect of ELA in opioid-naïve animals observed on independent t-test did not survive multiple post-hoc comparisons following ANOVA (t_(27)_=2.2881, P=0.06). Within the amygdala, these effects were unique to BLA: there was no effect of ELA (F_(1,27)_<0.01, P=0.94), nor an interaction of ELA with opioid experience in CeA (F_(1,27)_=0.1624, P=0.69). Instead, prior opioid exposure led to reduced cFos after heroin regardless of rearing condition (Fig 1E; F_(1,27)_=4.458, P=0.044).

Within the PFC (Fig. 1F-G), cFos in the prelimbic cortex (PLC) after heroin was also influenced by both ELA and opioid experience. Opioid-naïve ELA rats had lower cFos expression than controls (t_(12)_=3.583, P=0.0038), but no such effect of ELA was observed in opioid-experienced animals (t_(15)_=0.2769, P=0.79). While opioid experience was associated with an overall reduction in cFos expression (F_(1,27)_=16.96, P=0.0003), a rearing condition x opioid experience interaction did not reach significance (F_(1,27)_=2.429, P=0.13; main effect of rearing F_(1,27)_=1.182, P=0.29). These effects were specific to PLC: in the infralimbic cortex (ILC), there was no effect of opioid experience or ELA on cFos levels following acute heroin (main effect of opioid experience F_(1,27)_=0.05294, P=0.82; rearing F_(1,27)_=2.712, P=0.11; interaction F_(1,27)_=0.1832, P=0.67).

In PVT (Fig. 1H), prior opioid experience reduced cFos expression after acute heroin in both ELA and CTL rats (F_(1,23)_=5.808, P=0.024), however ELA did not further impact neuronal activation in this region (F_(1,23)_=0.3054, P=0.59; interaction F_(1,23)_=0.2050, P=0.66).

**Fig. 2.**
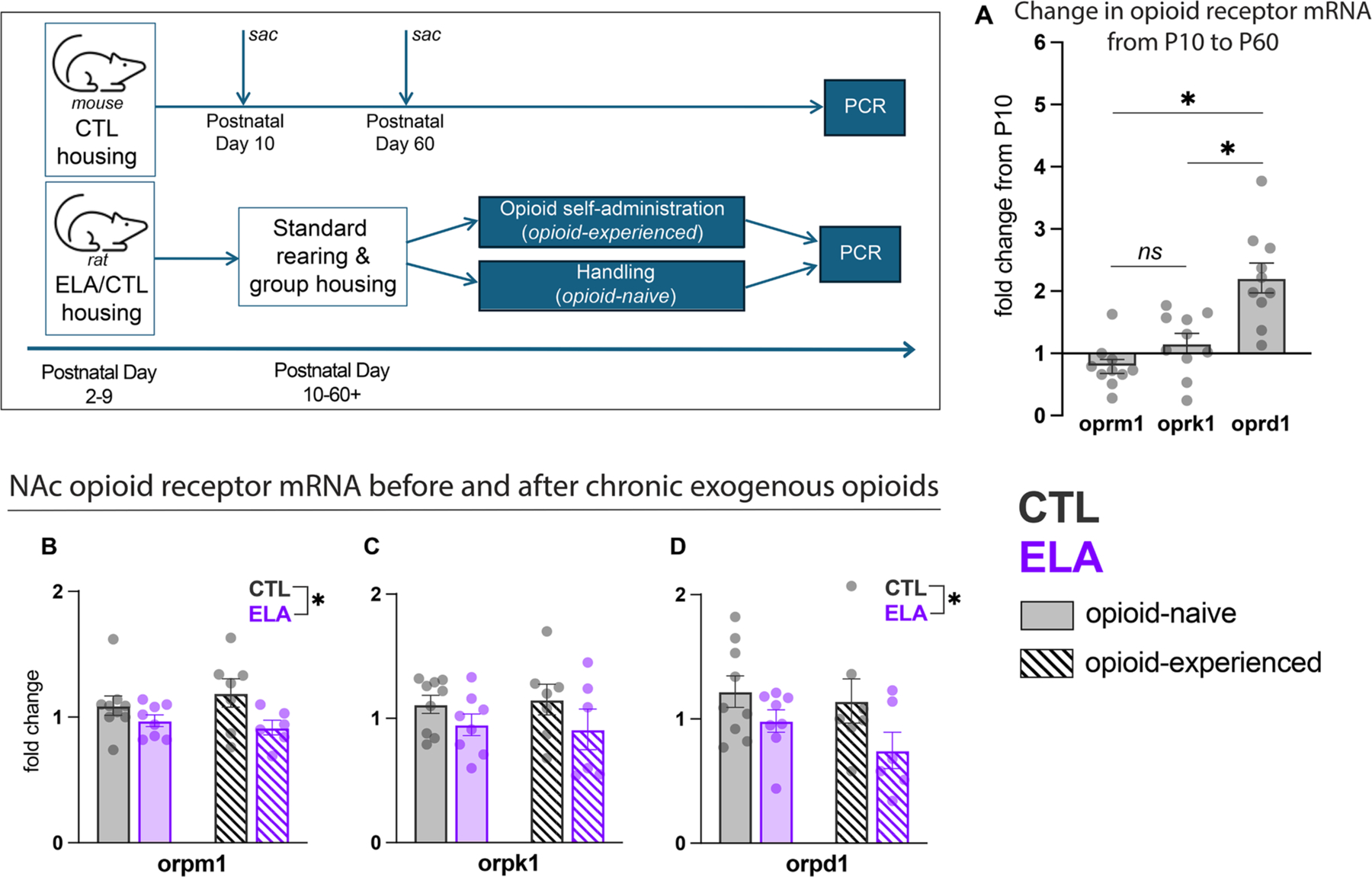
Developmental effects of ELA on opioid receptor mRNA expression. (A) In the mouse NAc, oprd1 had a larger-fold increase in expression by PD60 relative to PD10 levels than oprm1 and oprk1. PD60 mRNA levels represented as fold-change relative to PD10 mean, normalized to a value of 1. (B-D) ELA decreased expression of delta and mu opioid receptor mRNA in NAc, and tended to decrease kappa opioid receptor mRNA. Data represented as fold-change relative to an internal control. * P<0.05

We also asked whether NAc-projecting neurons may play a role in mediating heroin-associated cFos effects observed in NAc, however, we did not observe any significant effects of ELA on cFos expression in NAc-projecting (CTb+) neurons within the regions we examined (Fig. 1I-M). In BLA (Fig. 2J), ELA and opioid history seemed to impact NAc-projecting BLA cells similarly to how all BLA cells were affected, in that ELA trended toward increasing cFos in CTb+ cells only in opioid naïve animals (interaction: F_(1,23)_=3.189, P=0.087; main effect of opioid experience: F_(1,23)_=0.7222, P=0.40; main effect of rearing: F_(1,23)_=0.01963, P=0.89).

Prior opioid experience was also associated with fewer active NAc-projecting cells within prelimbic PFC (PLC), but no effect of ELA was seen (Fig. 1K; opioid experience: F_(1,22)_=9.947, P=0.0046, rearing: F_(1,22)_=1.558, P=0.23; interaction: F_(1,22)_<0.01, P=0.92). Activation of NAc-projecting cells within infralimbic PFC (ILC) was not affected by either ELA or opioid experience (Fig. 1L; rearing: F_(1,22)_=0.2424, P=0.63; opioid experience: F_(1,22)_=1.026, P=0.3220; interaction: F_(1,22)_=0.1545, P=0.70).

Finally, heroin in opioid experienced rats yielded fewer active NAc- projecting PVT cells, with no influence of ELA history (Fig. 1M; opioid experience: F_(1,20)_=18.55, P<0.001; rearing F_(1,20)_=0.2384, P=0.63; interaction F_(1,20)_<0.01, P>0.9).

### 3.2. Effects of ELA on NAc opioid receptor mRNA expression

Given the robust effects of ELA on NAc responses to opioid drugs described above, we next asked whether changes in NAc opioid receptor expression may be a mechanism by which this occurs. First, we sought to determine whether opioid receptors may be especially vulnerable to disruptions early in life, and did so by quantifying postnatal changes in their expression under control rearing conditions. In NAc, we measured mRNA transcripts of mu, kappa, and delta opioid receptors (*oprm1, oprk1, and oprd1, respectively)* just after the first week of life (PD10), and compared these levels to the levels of the same transcripts seen at maturity (PD60). In control female mice, we found that *oprd1* expression was still immature on PD10, in accord with prior work [80,81]. The change in *oprd1* levels by P60 was significantly greater than changes in *oprm1* and *oprk1* (Fig. 2A; One-way ANOVA F_(2,27)_=17.14, P<0.0001; post-hoc multiple comparisons: *oprd1* vs. *oprm1* t_(27)_=5.644, P<0.0001, *oprd1 vs. oprk1* t_(27)_=4.170, P=0.0008; *oprm1 vs oprk1:* t_(27)_=1.473. P=0.39). Additionally, *oprd1* expression at PD10 was significantly lower than at PD60 (t_(12)_=3.023, P=0.011), whereas *oprm1* and *oprk1* at PD10 had already reached mature PD60 levels (*oprm1:* t_(12)_=1.170, P=0.26; *oprk1:* t_(12)_=0.52344, P=0.61). Therefore, we hypothesized that ELA may have a greater ability to disrupt delta opioid receptors due to their late postnatal development relative to the other major opioid receptors.

To test this, we next measured opioid receptor mRNA in NAc of control and ELA-exposed animals. Given that ELA and CTL rats had differential cFos expression in response to heroin based on prior opioid self-administration experience, we also tested whether opioid self- administration experience affected the mRNA levels of these receptors (Fig. 2B-D). We found that ELA rats had lower NAc expression of mu and delta opioid receptor mRNA than controls (*oprm1* and *oprd1*, respectively; main effect of rearing *oprm1:* F_(1,26)_=6.279, P=0.02; *oprd1:* F_(1,26)_=5.361, P=0.0297). There was a similar but nonsignificant trend for kappa opioid receptor as well (*oprk1*; F_(1,26)_=3.481, P=0.070). Chronic opioid self-administration experience did not affect NAc mRNA for any of the three receptors (opioid experience: *oprm1* F_(1,26)_=0.0843, P=0.77; *oprk1* F_(1,26)_<0.01, P>0.99; *oprd1* F_(1,26)_=1.305, P=0.26. Interaction: *oprm1* F_(1,26)_=0.9675, P=0.33; *oprk1* F_(1,26)_=0.1246, P=0.73; *oprd1* F_(1,26)_=0.3474, P=0.56).

### 3.3. Effects of NAc DOR activation and blockade on demand for self- administered opioids

**Fig. 3.**
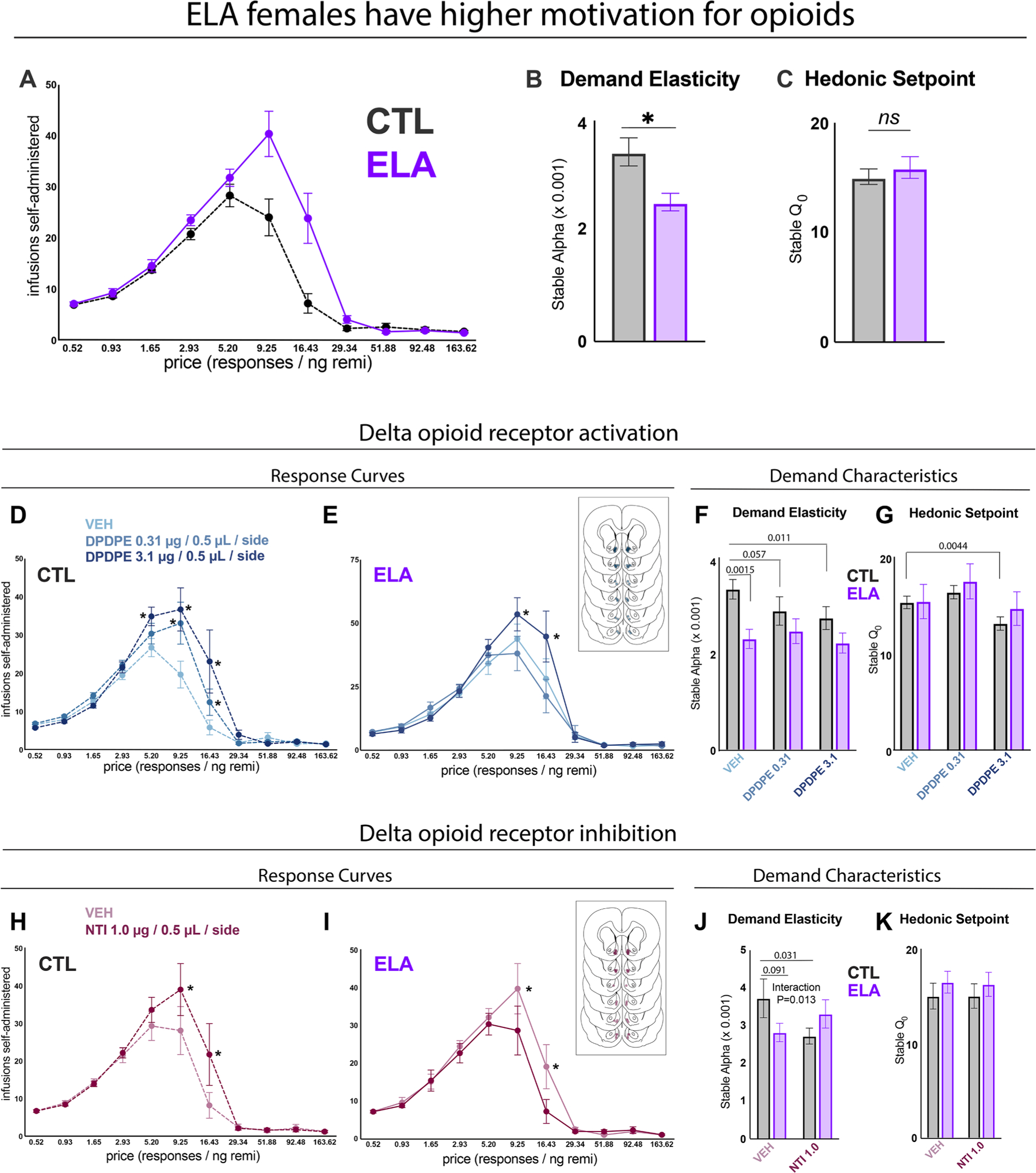
Pharmacological manipulation of NAc DORs recapitulates ELA phenotype. (A) Vehicle test day remifentanil consumption (infusions) at each price point on the within-session behavioral economic demand task. Infusions at low price points represent larger doses per infusion. As price increases, infusion volume decreases, thus reducing the dose and leading to increasing infusions at higher price points until a maximal effort is reached, at which point the number of infusions self-administered decreases. (B-C) demand characteristics generated from fitted demand curves (fitted curves not shown). ELA females consumed more remifentanil at high price points and had reduced demand elasticity (lower α; increased motivation) relative to controls. The animals did not differ on hedonic setpoint (Q_0_). (D) NAc DOR stimulation with DPDPE significantly increased consumption in higher-price bins in CTL rats. (E) Only high-dose DPDPE increased consumption in ELA rats. (D) ELA rats had lower demand elasticity than CTLs, as expected, and high-dose DPDPE significantly reduced demand elasticity in CTL rats. (G) High dose DPDPE also reduced hedonic setpoint in CTL rats. (H) NAc DOR inhibition with NTI increased responding in higher-price bins in CTL rats, (I) and reduced high-price responding in ELA rats. (J) NTI decreased demand elasticity in CTL rats but did not significantly alter demand elasticity in ELA rats, despite reduced high-effort responding. (K) NTI had no effect on hedonic setpoint in either ELA or CTL rats. *Agonist/antagonist dose vs. vehicle: P<0.05

Since we found that ELA downregulated delta opioid receptor mRNA and altered NAc responses to heroin in both opioid-naïve and opioid-experienced females, we hypothesized that persistent changes in endogenous delta opioid receptor signaling in NAc may mediate ELA-augmented opioid seeking in female rats [28]. We previously reported that ELA increases motivation for remifentanil in a behavioral economic task, as reflected by reduced demand elasticity [28]. Here, we replicated key finding (Fig. 3A-C; t_(49)_=3.156, P=0.0027), and also confirmed that ELA does not impact low-effort intake of remifentanil, or “hedonic set-point” for the drug (t_(49)_=0.6764, P=0.50). To test the role of NAc delta opioid receptors in ELA-augmented, effortful opioid demand, we pharmacologically stimulated and inhibited delta opioid receptors with infusions of agonist and antagonist drugs into the NAc medial shell prior to behavioral economic demand elasticity tests.

#### 3.3.1 Effects of NAc DOR stimulation

Activating DORs with the agonist DPDPE led to increased high-effort remifentanil consumption, and thus reduced demand elasticity, in a dose-dependent manner, mimicking an ELA-like phenotype. Specifically, in CTL rats (Fig. 3D), DPDPE significantly increased responding in high-price bins during the within-session demand task (DPDPE dose F_(2,22)_=5.312, P=0.013; price F_(10,110)_=52.16, P<0.0001; interaction F_(20,220)_=3.827, P<0.0001). Post-hoc comparisons showed that DOR stimulation with high dose DPDPE elevated responding relative to VEH in bins 5-7, the highest-price bins before which responding approached zero (price [responses/ng remifentanil] = 5.2: P=0.0052; 9.25: P<0.0001; 16.43: P<0.0001). Stimulation with low dose DPDPE also significantly increased responding at price = 9.25 (P<0.0001) and 16.43 (P=0.029). In contrast, high dose DPDPE in ELA rats (Fig. 3E) only modestly increased responding at select high-price bins. Although the main effect of DPDPE dose did not reach significance in the ELA group (F_(2,22)_=2.894, P=0.077), there was a significant effect of price (F_(10,110)_=37.88, P<0.0001) and dose x price interaction (F_(20,220)_=2.960, P<0.0001). Post-hoc multiple comparisons showed that DOR stimulation only with high dose DPDPE elevated responding relative to VEH at price = 9.25 (P=0.015) and 16.43 (P<0.0001) in ELA females. Stimulation with low dose DPDPE did not alter responding at any price in ELA rats.

In accordance with increasing high-price responding in CTL rats, DOR stimulation with DPDPE also dose-dependently decreased demand elasticity for remifentanil across the session (α; Fig. 3F). One-way repeated measures ANOVA revealed a significant effect of treatment dose in CTL animals, with a significant reduction in demand elasticity (α) at the highest dose of agonist relative to vehicle, and a near-significant reduction with the lower dose (F_(2,22)_ = 5.054, P=0.016; vehicle vs. DPDPE 0.31 P=0.057; vehicle vs. DPDPE 3.1 P=0.011). DOR stimulation in ELA animals did not significantly alter demand elasticity (F_(2,22)_=0.7410, P=0.49). While we observe distinct effects of DOR stimulation in ELA and CTL animals, the trending interaction of rearing condition and DOR stimulation did not reach significance (interaction F_(2,44)_=2.664, P=0.0809; rearing F_(1,22)_=5.113, P=0.034; DPDPE dose F_(2,44)_=2.930, P=0.064).

In both ELA and CTL rats, stimulation of DORs with DPDPE also altered hedonic setpoint (*Q*_*0*_; Fig. 3G; CTL: F_(2,22)_=13.54, P<0.001; ELA: F_(2,22)_=4.577, P=0.022). Post hoc tests revealed that in CTL rats, treatment with the high agonist dose resulted in lower hedonic setpoint relative to vehicle (P=0.0044), whereas post hoc tests did not reach significance in ELA rats (VEH vs DPDPE 0.31 P=0.076, VEH vs. DPDPE 3.1 P=0.67). The interaction between rearing condition and DPDPE dose was not significant (interaction F_(2,44)_=0.8373, P=0.44; rearing F_(1,22)_=0.2660, P=0.61; DPDPE dose F_(2,44)_=13.73, P<0.0001).

#### 3.3.2. Effects of NAc DOR inhibition

In control rats, blocking NAc DORs increased remifentanil consumption at higher effort requirement bins. There was a significant main effect of price (Fig. 3H; F_(10,90)_=23.55, P<0.0001), a near-significant effect of NTI (F_(1,9)_=4.703, P=0.058), and significant NTI x price interaction (F_(10,90)_=2.677, P=0.0065). Post-hoc tests revealed that consumption was significantly higher after DOR inhibition at price = 9.25 (P=0.0014) and price = 16.43 (P=0.0032).

Conversely, in ELA rats, blockade of NAc DORs significantly reduced consumption at higher-effort bins. There was a significant main effect of price (Fig. 3I; F_(10,150)_=29.35, P<0.0001), NTI (F_(1,15)_=4.613, P=0.049), and a significant interaction of price with NTI dose (F_(10,150)_=2.580, P=0.0065). DOR inhibition reduced consumption at price = 9.25 (P=0.0016) and price = 16.43 (P=0.0006).

Aligning with the effects of DOR antagonism on high-effort consumption, NTI significantly decreased demand elasticity (α) in CTL rats (t_(10)_=2.512, P=0.031). While the effect of NTI on demand elasticity in ELA rats was not significant (t_(10)_=1.314, P=0.21), there was a significant rearing x NTI interaction on demand elasticity, supporting the notion that ELA and CTL animals may respond differently to DOR blockade (Fig. 3J; F_(1,25)_=7.096, P=0.013; rearing: F_(1,25)_=0.1487, P=0.70; NTI: F_(1,25)_=0.8197, P=0.37). There were no observed effects of ELA or NTI on hedonic setpoint (*Q*_*0*_; Fig. 3K; rearing: F_(1,25)_=0.6443, P=0.43; NTI: F_(1,25)_<0.1, P=0.87; interaction: F_(1,25)_<0.1, P=0.86).

### 3.4. Effects of ELA on the endogenous opioid system in other reward circuit nodes

**Fig. 4.**
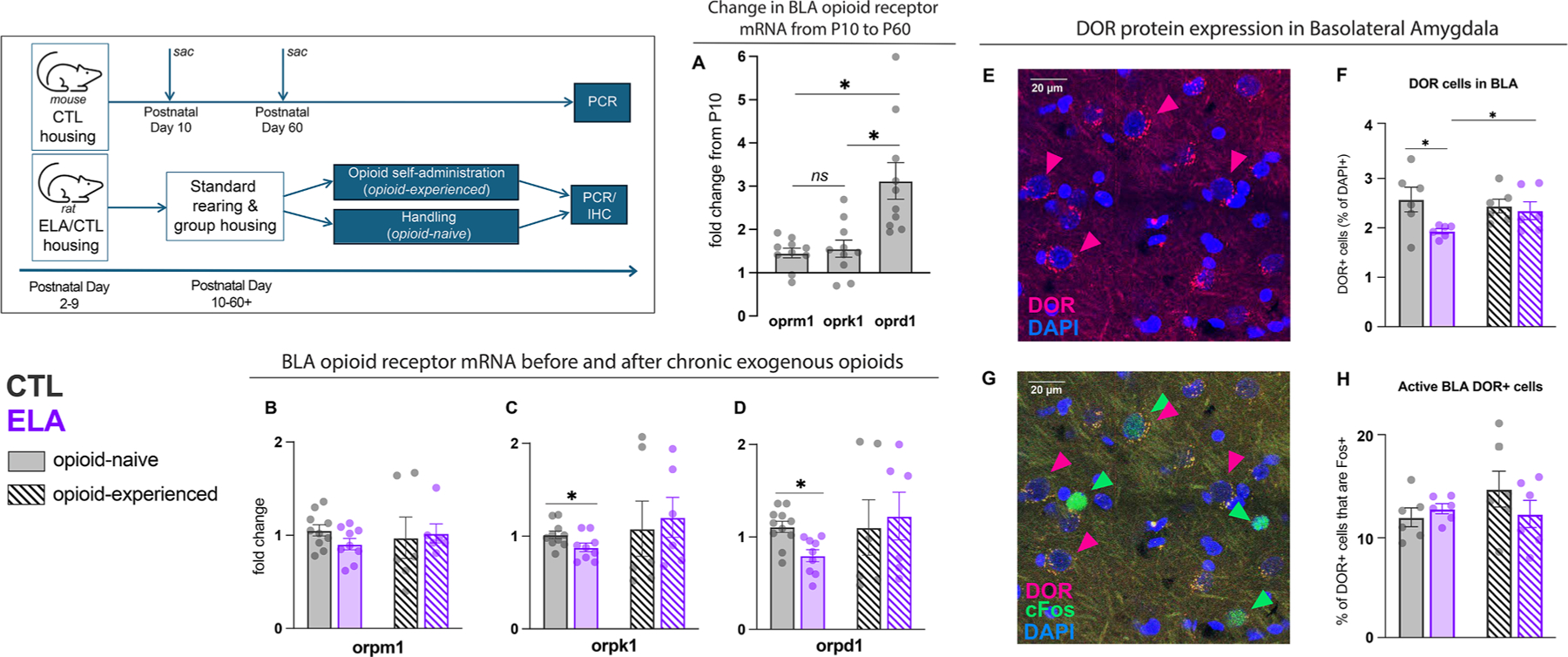
ELA alters DOR expression in BLA. (A) In the mouse BLA, a developmental expression profile was noted for oprd1 but not oprm1 or oprk1. Specifically, relative to PD10 levels (normalized as 1) oprd1 were three-fold higher by PD60, while the increase in oprm1 and oprk1 was minimal. PD60 mRNA levels represented as fold-change relative to PD10 mean, normalized to a value of 1. (B-D) In BLA, *oprd1* and *oprk1* were significantly reduced after ELA only in opioid naïve animals, and neither ELA nor opioid experience affected *orpm1* expression in BLA. Data represented as fold-change relative to an internal control. (E) Representative image of DOR+ cells in BLA. (F) Opioid-naïve, ELA-reared females had fewer DOR+ cells in BLA compared to opioid-naïve controls, mirroring *oprd1* expression in the region. Following opioid experience, DOR protein expression returned to control levels. (G) Representative cFos+/DOR+ cells in BLA. (H) Although ELA altered DOR expression in BLA, there was no difference in the activation of DOR+ neurons between ELA and CTL animals. *P<0.05

In addition to the effects of ELA on the NAc described above, we also found a robust effect of ELA on heroin-induced cFos expression within BLA. Therefore, we hypothesized that ELA might affect opioid receptor levels in this structure in a manner similar to that observed in NAc. Accordingly, we also hypothesized that BLA DORs would reach mature levels later in life, akin to our findings in NAc. Indeed, the change in *oprd1* levels in BLA of control female mice by P60 was significantly greater than changes in *oprm1* and *oprk1* (Fig. 4A; One-way ANOVA F_(2,27)_=11.37, P=0.0003; post-hoc multiple comparisons: *oprd1 vs oprm1* t_(27)_=4.252, P=0.0007, *oprd1 vs. oprk1* t_(27)_=3.997, P=0.0013, *oprm1 vs. oprk1* t_(27)_=0.2549, P=0.99), and *oprd1* expression was significantly lower at PD10 than PD60 (t_(12)_=3.018, P=0.011). *oprm1* was also significantly lower at PD10 compared to PD60, and *oprk1* was not different at PD10 and PD60 (*oprm1:* t_(12)_=2.200, P=0.048; *oprk1:* t_(12)_=1.572, P=0.14). Similar to our findings in NAc, in BLA of adult female rats, ELA animals had decreased kappa and delta opioid receptor mRNA levels. Interestingly, chronic exposure to opioids appeared to normalize these ELA-induced changes (Fig. 4C-D; *oprk1:* naïve t_(17)_=2.158, P=0.046; experienced t_(10)_=0.3358, P=0.74; *oprd1*: naïve t_(18)_=3.476, P=0.0027; experienced t_(10)_=0.3125, P=0.76), though the overall ELA/opioid experience interaction did not reach significance (*oprk1:* interaction F_(1,27)_=0.7599, P=0.39; opioid experience F_(1,27)_=1.175, P=0.20; rearing F_(1,27)_<0.01; P>0.9; *oprd1*: interaction F_(1,28)_=1.833, P=0.19; opioid experience F_(1,28)_=1.722, P=0.20; rearing F_(1,28)_=0.3424; P=0.56). *oprm1* expression in BLA was not affected by ELA in either opioid-naïve or opioid experienced rats (Fig. 4B; naïve: t_(17)_=1.724, P=0.10; experienced t_(10)_=0.1981, P=0.85), and there was also no effect of opioid experience or ELA/opioid experience interaction (interaction F_(1,27)_=0.8002, P=0.38; opioid experience F_(1,27)_=0.0277, P=0.87; rearing F_(1,27)_=0.2051; P=0.65).

To determine whether opioid receptor expression was altered specifically in heroin-responsive cells in BLA, we immunolabeled delta opioid receptor protein along with cFos. Delta opioid receptor protein expression results largely recapitulated ELA effects on *oprd1* mRNA, confirming protein-mRNA congruency [64,82]. Opioid-naïve ELA rats had fewer DOR+ cells in BLA than CTL rats (Fig. 4F; t_(10)_=2.515, P=0.031), an effect that was not observed in rats with chronic opioid experience (t_(10)_=0.4177, P=0.69). Further recapitulating mRNA results, opioid experience seemingly normalized DOR protein expression in ELA rats, as opioid experienced ELA rats had significantly more DOR+ cells than opioid naïve ELA rats (t_10_=2.219, P=0.05). We note however that the overall interaction of ELA and opioid experience did not reach statistical significance (interaction F_(1,20)_=2.570, P=0.12; rearing condition F_(1,20)_=4.656, P=0.043; opioid experience F_(1,20)_=0.6877, P=0.42). Fluorescently-labeled cFos expression measured here followed a similar pattern to our prior experiment, with a trending interaction of rearing condition and opioid experience (F_(1,20)_=3.456, P=0.078; main effect of opioid experience: F_(1,20)_=4.464, P=0.047; main effect of rearing: F_(1,20)_=1.318, P=0.26). Though ELA altered both delta opioid receptor and cFos expression in BLA, these cells did not appear to comprise the same population. ELA rats did not differ from controls in the activation of DOR-expressing cells specifically, as measured by percentage of DOR+ cells that also expressed cFos (Fig. 4H; opioid--naïve: t_(10)_=0.8065, P=0.44; opioid-experienced: t_(10)_=1.107, P=0.29).

To test whether the effects of ELA on opioid receptor expression are selective to only certain nodes within reward circuits, we also measured opioid receptor mRNA in PVT and PFC, structures in which we did *not* observe ELA-induced changes in their response to heroin. Neither ELA nor opioid experience impacted PVT opioid receptor mRNA levels (Supplemental Fig. 1A; opioid experience: *oprm1* F_(1,26)_=0.084, P=0.77; *oprk1* F_(1,26)_<0.01, P>0.99; *oprd1* F_(1,26)_=1.305, P=0.26. Interaction: *oprm1* F_(1,26)_=0.9675, P=0.33; *oprk1* F_(1,26)_=0.1246, P=0.73; *oprd1* F_(1,26)_=0.3474, P=0.56). In PFC, ELA did not affect receptor mRNA, though *oprm1* and *oprd1* levels were increased after chronic opioid experience (Supplemental Fig. 1B; opioid experience: *oprm1* F_(1,28)_=6.230, P=0.0187; *oprk1* F_(1,28)_=0.8179, P=0.37; *oprd1* F_(1,28)_=7.872, P=0.0090. Rearing: *oprm1* F_(1,28)_=0.6475, P=0.43; *oprk1* F_(1,28)_=0.0645, P=0.80; *oprd1* F_(1,28)_=0.4985, P=0.85. Interaction: *oprm1* F_(1,28)_=0.8431, P=0.37; *oprk1* F_(1,28)_=0.5447, P=0.47; *oprd1* F_(1,28)_=0.1460, P=0.71).

Finally, since we previously found that ELA has markedly sex-dependent effects on reward behaviors in males and females [28,30], we sought to determine whether opioid receptor mRNA findings were specific to females, in whom we observe a pro-opioid phenotype. We thus measured *oprm1, oprk1, and ord1* mRNA in NAc and BLA of opioid-naïve male rats, and found that ELA did not impact any of the transcripts relative to control males (Supplemental Fig. 1C-D; NAc: *oprm1* t_(14)_=0.8703, P=0.40; *oprk1* t_(14)_=0.2770, P=0.79; *oprd1* t_(14)_=0.4180, P=0.68. BLA: *oprm1* t_(14)_<0.1, P>0.9; *oprk1* t_(14)_=0.6005, P=0.56; *oprd1* t_(14)_=1.701, P=0.11.)

## 4. Discussion

The data presented here support a novel role for DOR signaling in the long-lasting impact of ELA on opioid addiction-like behaviors in female rats. We find that both the expression and behavioral function of DORs are altered by ELA, effects that are further modulated by subsequent opioid exposure in adulthood. These findings implicate DORs in ELA-induced behavioral phenotypes in female rats, and provide an impetus for further research into their roles in the fundamental mechanisms of addiction, as well as their potential targeting in future addiction prevention and treatment strategies.

### 4.1. Impacts of ELA on heroin-induced neuronal activation with or without prior opioid experience

We previously reported that ELA leads to sex-dependent effects on reward behaviors and associated neural activity [28,30]. To better understand the specific ELA-induced changes responsible for the pro-addiction phenotype we see in female rats, we screened several reward circuit nodes for differences in their responses to heroin. We found that in NAc, ELA females receiving their first ever heroin injection as adults displayed markedly reduced cFos expression relative to control-reared counterparts. These findings were especially robust in the dorsomedial shell subregion, an area known to contain an opioid “hedonic hotspot” [42,79], suggesting that this zone may be a particular target of ELA-induced reward dysfunction. However, after several weeks of opioid self-administration, ELA females had increased NAc cFos relative to controls—an apparent reversal of the pattern observed in opioid-naïve groups. Intriguingly, this reversal appears to be driven by a change in cFos expression in *control* animals, raising the possibility that ELA rats lack a compensatory mechanism that may otherwise protect against compulsive drug seeking.

In contrast to NAc, BLA heroin-induced cFos was enhanced in opioid-naïve ELA rats compared to controls, a difference that appeared to normalize following chronic opioid self-administration. Similarly, in prelimbic cortex, heroin-induced cFos expression was lower in opioid-naïve ELA rats than controls, and this difference was also no longer apparent after opioid self-administration. These structures may therefore be particularly responsive to early opioid experiences of ELA animals. Indeed, it is worth noting that individuals who develop substance use disorders tend to report a more salient subjective experience of their first use, compared to those who use drugs but do not meet criteria for a substance use disorder [83–85]. Thus, understanding how ELA impacts reward circuit responses to opioids during the first drug experience may provide insights into the progression to addiction that may develop differently in those with a history of adversity.

In addition, we found that chronic opioid experience suppressed heroin-induced cFos in prelimbic PFC and PVT of both ELA and control rats, suggesting that these regions may play a more general role in encoding drug experiences, consistent with existing literature [47,48, 51,86,87].

Finally, we also examined heroin-associated cFos expression in NAc-projecting neurons of BLA, PLC, ILC, and PVT that provide addiction-relevant, predominantly glutamatergic inputs. Neither ELA nor opioid experience consistently altered cFos in these identified projection neurons. This suggests that other NAc-projecting neuronal populations, or possibly subsets of neurons within these regions that comprise a smaller NAc-projecting microcircuit not detectable with CTb retrograde tracing alone [88], may instead be implicated in the overall effects of ELA on NAc cFos expression after acute heroin. The nature and connectivity of this population should be further investigated.

### 4.2. NAc opioid receptors as a target of ELA

To further explore the mechanistic changes that may underlie the persistently disrupted NAc responses to heroin observed after ELA, we sought to characterize ELA-induced changes in the function of the endogenous opioid system within this structure. Exploring the developmental trajectory of opioid receptors in NAc of control animals, we found that delta opioid receptor mRNA levels had not yet reached that of the adult by the end of the early postnatal period (postnatal day 10), whereas mu and kappa opioid receptors had already reached maximal expression. This is consistent with prior reports in rats and mice that have suggested that delta, relative to mu or kappa opioid receptors, reach mature levels of expression during the early postnatal period in which we conduct our limited bedding and nesting ELA protocol [80, 81]. In adult female rats, delta opioid receptor expression was suppressed in ELA females both before and after opioid self-administration experience (a similar pattern was also observed with mRNA for MOR and KOR). From these results, it is possible to hypothesize that delta opioid receptors may be uniquely susceptible to the developmental effects of ELA, and that ELA-induced changes in their expression represent a vulnerable state within brain circuitry that predispose an individual to harmful substance use or addiction [89].

Taken together, the data described above set up the possibility that delta opioid receptors may play a causal role in the pro-opioid addiction phenotype we previously observed [28]. To test this, we employed pharmacological manipulations of DORs using intra-accumbens microinjections of the selective DOR agonist DPDPE, or the selective antagonist NTI, in ELA- or control-reared females. Replicating our prior finding using a behavioral economic demand protocol [28], we observed that ELA females, relative to controls, had enhanced motivation to pursue remifentanil (lower demand elasticity, α), but did not differ in their seeking at low effort (“hedonic setpoint”, Q_0_). Intriguingly, pharmaco- logic stimulation of delta opioid receptors dose-dependently increased motivation for remifentanil in control rats, recapitulating the ELA phenotype. Conversely, delta opioid receptor *blockade* in ELA rats *decreased* high-effort responding (decreased motivation), a partial rescue of the ELA phenotype. Curiously, DOR blockade modestly increased high-effort responding in control rats, as did DOR stimulation with high-dose agonist in ELA rats. Taken together, these results implicate delta opioid receptors in motivated drug seeking, and suggest that ELA may alter the manner in which DORs regulate this behavior.

Several mechanisms could underlie the behavioral effects of NAc DOR manipulations. We found that in control-reared rats both stimulation and blockade of DORs enhances motivation for opioids. One possible explanation of this is that two separate NAc DOR populations are being targeted by the agonist and antagonist. For example, DORs and other opioid receptors are present both pre- and post-synaptically [46, 90–93] and thus stimulation and blockade of these receptors may lead to similar outcomes depending on the output of downstream signaling cascades within in the region.

In contrast, blocking DORs in ELA-reared rats, in whom DOR expression is already reduced relative to control-reared rats, partially rescues the ELA opioid seeking phenotype. It is worth noting that we observed some significant and near-significant changes in MOR and KOR mRNA expression in our structures of interest. Thus, it is possible that ELA-induced changes in the interactions between DORs and other colocalized receptors may play a role in the behaviors we observe. Indeed, DORs are known to form heteromeric complexes with MORs and KORs that produce diverse functional consequences [94–97], and the expression of these heteromers might also be mediated by chronic opioid exposure [98]. ELA-induced changes to these receptor interactions may therefore cause the unexpected reversal of the pro-opioid phenotype in response to DOR blockade, despite already reduced levels of the receptor—though this possibility remains to be tested.

Finally, DOR receptors have previously been shown to localize preferentially to cholinergic interneurons within the NAc, which represents another possible mechanism by which DORs may indirectly affect neuronal reward signaling in this region in both ELA and control animals [45,46,92,99]. For example, ELA may disrupt the typical inhibitory tone provided by these interneurons, leading indirectly to enhanced reward seeking. This may be further perturbed by chronic opioid use, such that DOR blockade permits normal function within these microcircuits and therefore reduces motivation for opioids in ELA rats.

Overall, it is clear that additional work to further characterize the identity of the DOR-expressing cells affected by ELA, as well as the role of DORs and their region-specific interactions with other opioid receptors and neural populations in control and ELA animals, is required to fully understand how ELA disrupts the endogenous opioid system.

### 4.3. Exploring functional consequences of ELA in other reward circuit nodes

In the above sections, we propose a role for delta opioid receptors specifically in the NAc in the dysregulation of reward seeking following early life adversity. Yet, we also found that ELA affected responses to opioid drugs in other circuit nodes. Therefore, we asked whether delta opioid receptors might play a role in these phenomena as well. In PFC and PVT, levels of delta, kappa, and mu opioid receptor mRNA transcripts were unchanged by ELA, in alignment with the minimal effects of ELA on cFos expression in these regions. In BLA, mRNA for mu and kappa opioid receptors were unaltered by either ELA or opioid history. In contrast, mRNA for DOR was markedly decreased in drug-naïve females with a history of ELA. With opioid experience, however, this ELA effect appears to have been normalized, with equivalent DOR expression now seen in ELA and control individuals. Furthermore, similar to NAc, mRNA levels for delta opioid receptors were still immature during early postnatal development, whereas mu and kappa receptor transcripts had already reached mature levels. These findings, especially in BLA, suggest that delta opioid receptors may be acting more broadly within limbic networks to mediate the effects of early life adversity on brain responses to opioid drugs.

To test the above hypothesis, we asked whether the distribution of delta opioid receptors might correlate with heroin-responsive cells in the BLA. We labeled tissue from adult opioid naïve and opioid experienced ELA and control rats who had received an acute injection of heroin (as in the above cFos experiments). We quantified cell-specific staining for delta opioid receptor protein, along with cFos protein, and DAPI to identify all cellular nuclei. Mirroring mRNA results in BLA, the number of DOR+ cells was reduced, relative to control-reared rats, in BLA of opioid naïve ELA rats—an effect that appeared to normalize following opioid self-administration. Notably, activation of DOR+ cells by heroin in BLA (as measured by cFos and DOR co-labeling) did not differ between ELA and control animals, suggesting that the differences in heroin-induced cFos expression observed after ELA are not specific to DOR+ cells. This points to a more indirect role, if any, of DORs in mediating the effects of ELA on the cellular response to heroin in this structure and its downstream targets, and implies that NAc DORs specifically may play a uniquely important role in mediating the effects of ELA on drug seeking. Further work should examine the subtypes of BLA cells expressing DORs that are most impacted by ELA, and whether such ELA-sensitive populations are causally involved in ELA-induced behavioral phenotypes. It is also important to note that the reduction of oprd1 mRNA levels is expected to render some DOR-expressing cells below the level of detection of IHC, which could lead to an apparent reduction of DOR+ neurons, and future experiments should examine the potential effects of ELA on delta opioid receptor transcription versus the expression of the receptor protein on the cell membrane.

## 5. Conclusions

The results presented here suggest a plausible mechanism by which ELA may impose vulnerability to opioid addiction specifically in females: changes in reward circuit DOR signaling. We demonstrate that ELA exerts its influence on neuronal activation in a structure-specific manner, and propose that changes in the expression and/or function of DORs in particular may underlie these effects. DORs may be more vulnerable than other opioid receptors to ELA because their expression seems to be in flux during the early-life period in which limited bedding and nesting is imposed, leading to potent effects on reward circuit development [17,21]. While DOR-specific compounds have thus far not been effective for pain or depression in clinical trials despite their apparent promise in preclinical models, our discoveries point to the need for further inquiry into the potential role of these receptors in reward-related behaviors, and how changes to their function within reward circuits may underlie long-lasting impacts of ELA.

## Supplementary Material

Supplemental Table 1

Supplemental Figure 1

## Data Availability

Data will be made available on request.
