# Supplementary material for: Opioid drug seeking after early-life adversity: a role for delta opioid receptors
Source: Addict Neurosci. Author manuscript; Available in PMC 2025 Sep 24. (PMC12456463; doi:10.1016/j.addicn.2024.100175)
Supplement: Supplemental Table 1 [file NIHMS2061727-supplement-Supplemental_Table_1.docx]

**Table 1. PCR primer sequences**

| **Gene (Rat)** | **Forward primer** | **Reverse Primer** |
| --- | --- | --- |
| 18S rRNA | 5’-GGATCCATTGGAGGGCAAGT | 5’-ACGAGCTTTTTAACTGCAGCAA |
| Oprd1 | 5’- TGTGCTATGGCCTCATGCTG | 5’- ATTGATGTCCACCAGCGTCC |
| Oprk1 | 5’- CGCCTTGACTGAATCCCAAC | 5’- TGTTCAGCCTCGATGGGTCC |
| Oprm1 | 5’- CAACCTCGTCCACGATCGAA | 5’- CCAGTTAGGGCAATGGAGCA |
| **Gene (Mouse)** | **Forward primer** | **Reverse Primer** |
| 18S rRNA | 5’- GGGAGCCTGAGAAACGGC | 5’- GGGTCGGGAGTGGGTAATTT |
| Oprd1 | 5’- GCTGGTGGACATCAATCGG | 5’- GCGTAGAGAACCGGGTTGAG |
| Oprk1 | 5’- CTCTCCGGCCATCCCTGTTA | 5’- GTTGCGGTCTTCATCTTCGTGTAT |
| Oprm1 | 5’- GTGTCTTCATCTTCGCCTTCATCA | 5’- CTTTTCTTTGGAGCCCGACAGC |
