# Supplementary material for: Opioid drug seeking after early-life adversity: a role for delta opioid receptors
Source: Addict Neurosci. Author manuscript; Available in PMC 2025 Sep 24. (PMC12456463; doi:10.1016/j.addicn.2024.100175)
Supplement: Supplemental Figure 1 [file NIHMS2061727-supplement-Supplemental_Figure_1.pdf]

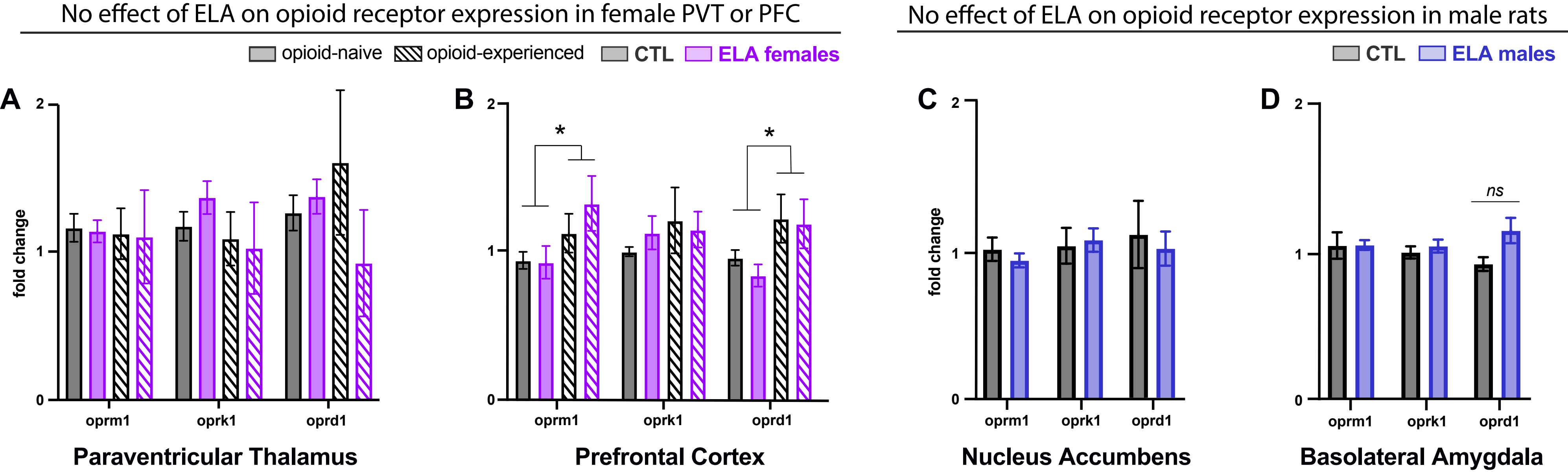

Supplementary Figure 1. ELA does not alter opioid receptor mRNA in PVT or PFC of females, NAc or BLA of males. (A) in PVT of female rats, there was no effect of ELA or opioid experience on opioid receptor mRNA levels. (B) In PFC, ELA and CTL female rats had higher levels of oprm1 and oprd1 than those without chronic opioid experience. (C, D). In BLA and NAc of male rats, ELA did not alter expression of opioid receptor mRNA. *P < 0.05.
